# Supplementary material for: Signature of type-II Weyl semimetal phase in MoTe2
Source: Nat Commun. 2017 Jan 13;8:13973. doi: 10.1038/ncomms13973 (PMC5241795; doi:10.1038/ncomms13973)
Supplement: Supplementary Information — Supplementary Figures 1-6 and Supplementary Notes 1-3 [file ncomms13973-s1.pdf]

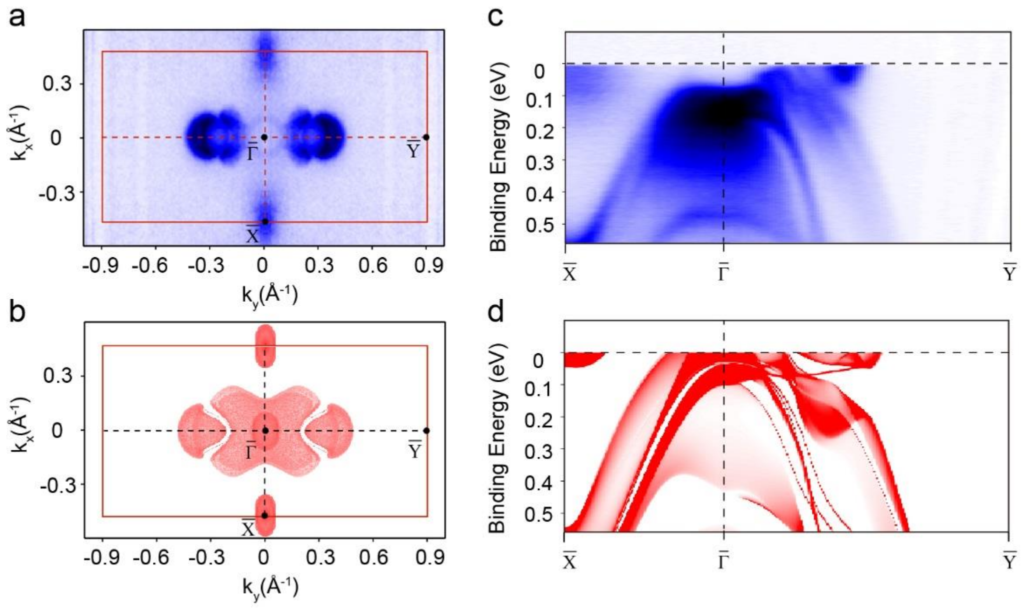

Supplementary Figure 1: Fermi surface and high-symmetry cut of $MoTe_2$ compared with the *ab-initio* calculation. a, the ARPES measured Fermi surface of $MoTe_2$. **b,** the calculated Fermi surface of $MoTe_2$. **c**, The band dispersion of $MoTe_2$ along $\bar{X} - \bar{\Gamma} - \bar{Y}$ obtained from ARPES measurement. **d,** The calculated band dispersion of $MoTe_2$ along $\bar{X} - \bar{\Gamma} - \bar{Y}$. This figure shows the Fermi surface mapping measured at a larger momentum scale compared to the one presented in the main text. One can clearly see that there are electron pockets around $\bar{X}$. This is in broad consistency with our *ab-initio* calculations.

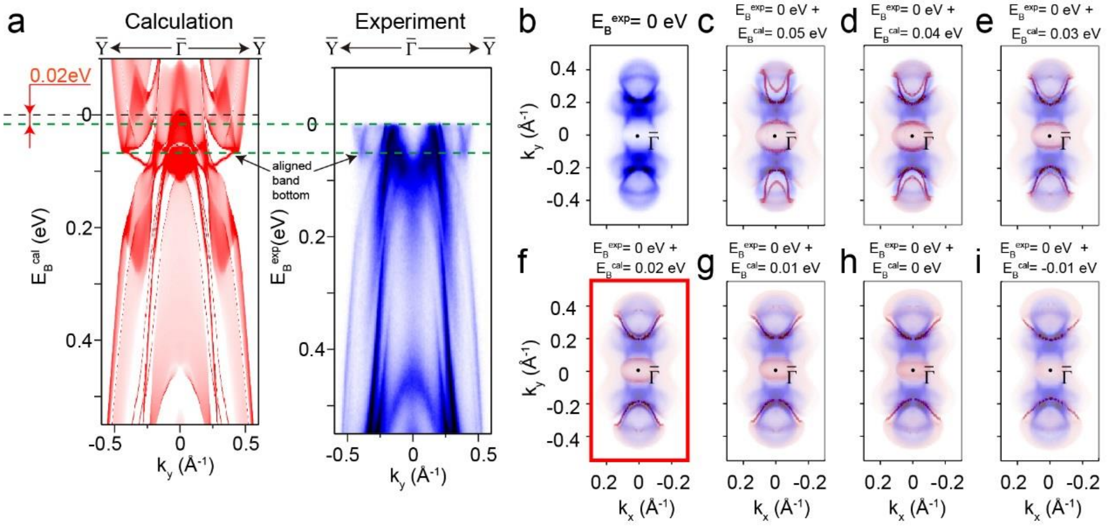

Supplementary Figure 2: Energy offset of the calculated and measured Fermi level of $MoTe_2$. **a**, comparison of the high symmetry cut along $\bar{Y}-\bar{\Gamma}-\bar{Y}$ between the calculation result and the experimental result. **b~i**, the measured Fermi surface of $MoTe_2$ compared with the calculated energy contours at different binding energies (E_B^{cal}). The red frame indicates the E_B^{cal} value which shows the best agreement.

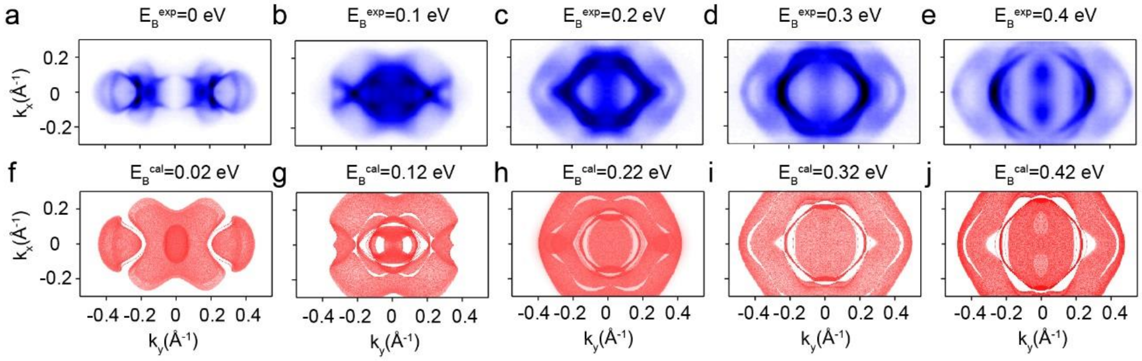

Supplementary Figure 3: Constant energy contours at different E_B compared with the ab-initio calculations. a~e, Constant energy contours at E_B^{exp}=0, 0.1, 0.2, 0.3, 0.4 eV, respectively. **f~j,** Constant energy contours at E_B^{cal}=0.02, 0.12, 0.22, 0.32, 0.42 eV, respectively.

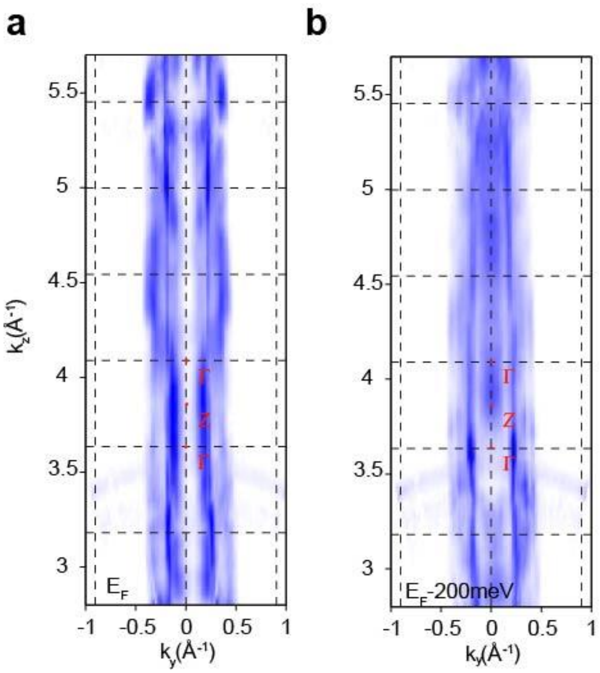

Supplementary Figure 4: k_z dependence of the electronic structure in $MoTe_2$. a, k_y-k_z map at the Fermi level. **b,** k_x-k_z map at 200 meV below the Fermi level. Here V_0=15.5 eV.

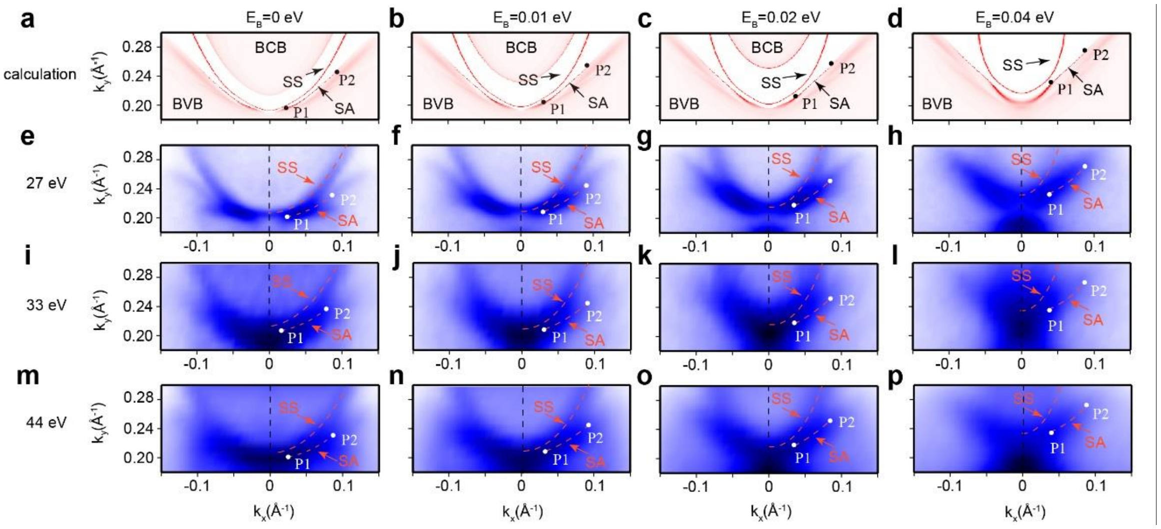

Supplementary Figure 5: Evolution of the Fermi arc state at different binding energies with various photon energies. a~d, Evolution of the projection of the Fermi arc states in the calculated constant energy contours at E_B=0, 0.01, 0.02 and 0.04 eV, respectively. **e-p,** The corresponding

photoemission intensity map in the same momentum range and binding energy as in **a** measured with 27 (**e~h**), 33 (**i~l**) and 44 eV (**m~p**) photons, respectively. The predicted features as in **a~d** are discovered and labelled. SS, surface state; SA: surface Fermi arcs state. P1 and P2: two terminals of the surface Fermi arc state. With photon energies ranging from 27 eV to 44 eV, the measured constant energy contours all show the signature of a surface state (SS) and the Fermi arc states (SA), consistent with the calculation, providing additional evidence of the existence of the Fermi arc states in $MoTe_2$.

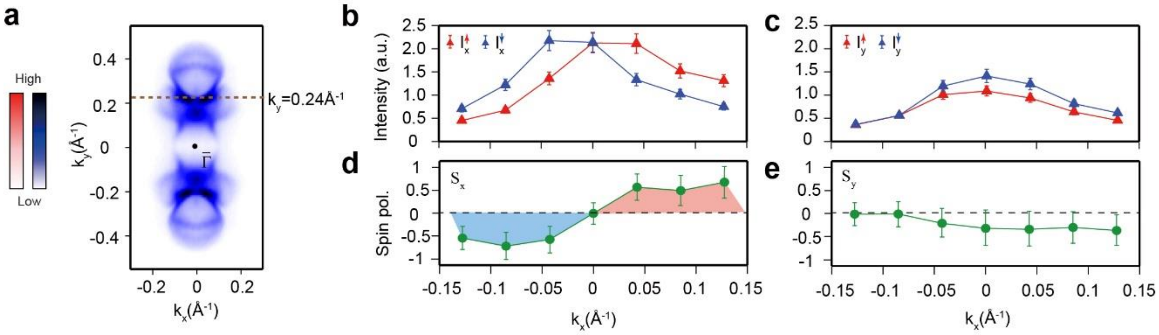

Supplementary Figure 6: Spin polarization of the surface arc states. a, The Fermi surface of the sample measured for spin-resolved ARPES, where the dashed line indicates the momentum positions where the spin-resolved measurements were performed. **b and c,** scattering intensity from two scattering targets orthogonal to each other. Red and blue symbols represent the scattering intensity with each scattering target when magnetized to up/down polarization, respectively. **d and e,** the asymmetry calculated along the k_x and k_y directions from **b** and **c** by $S_{x/y} = \frac{I^{Up}_{x/y} - I^{down}_{x/y}}{I^{Up}_{x/y} + I^{down}_{x/y}} / \eta$, respectively (where $\eta \approx 0.3$ is the Sherman function of the spin-detector), showing clearly the switching of sign for S_x (**d**) while not for S_y (**e**).

Supplementary Note 1: Comparison of the experimental results to theory

The detailed band structure and Fermi surface topology of $MoTe_2$ are compared with the theoretical calculations summarized in Supplementary Fig. 2. The band dispersion along $\bar{Y} - \bar{\Gamma} -$

$\bar{Y}$ is plotted in Supplementary Fig. 2a, where the left panel is the calculation result and the right panel is the experimental result. In order to fit the calculation with the experimental results, the Fermi level (E_F) in the calculation should be shift downward ~0.02 eV (here we use the band bottom of the electron bands as the alignment reference, as indicated in Supplementary Fig. 2a). Since the Weyl points in the calculation is 6 meV and 59 meV above E_F, thus in our data, the Weyl points should be further above E_F.

We also determined the energy offset from comparing the calculated and measured constant energy contours. Supplementary Fig. 2b shows the Fermi surface mapping of $MoTe_2$ measured with 27 eV photons. In Supplementary Fig. 2c~2i, the calculated constant energy contours at different binding energies (E_B^{cal}) are overlapped with the experimental Fermi surface (E_B^{exp}=0 eV) in Supplementary Fig. 2b. It is clear that the experimental result shows best consistency with the calculated result at E_B^{cal}=0.02eV, in agreement with the offset in Supplementary Fig. 2a.

To further elucidate the consistency between the experimental and calculated results, we plot the measured constant energy contours at different binding energies E_B^{exp}=0, 0.1, 0.2, 0.3 and 0.4 eV (Supplementary Fig. 3a~3e), together with the calculated constant energy contours at corresponding binding energies by taking into account the energy offset (0.02eV) at E_B^{cal}=0.02, 0.12 , 0.22, 0.32 and 0.42 eV (Supplementary Fig. 3f~3j). The evolution of the spectra shows well agreement.

Supplementary Note 2: Photon energy dependent measurements on the electronic structure of $MoTe_2$

ARPES measurement can determine the in-plane momentum ($k_{//,}$ parallel to the sample surface) of electrons in solids naturally by the momentum conservation of photoelectrons; while determining the out-of-plane momentum component (k_z) is less straightforward - which requires a set of ARPES measurements performed under different photon energies.

Based on the nearly free-electron final state approximation with a potential parameter V_0 (also known as the inner potential) describing the energy difference from the bottom of the final state band to the vacuum level, we can derive the k_z as:

$$k_z = \sqrt{\frac{2m_e{}^*}{\hbar^2}(E_k \cos^2\theta + V_0)}$$

where θ is the emission angle, m_e* is the effective mass of electrons in the final bulk states and E_k is the kinetic energy of the emitted free electron, which satisfies:

$$E_k = \mathrm{h}\upsilon - w - E_B$$

where $h\nu$ is the photon energy, w is the work function of the sample and E_B is the electron binding energy .

As V_0 is material dependent, we performed energy dependent ARPES by using a broad range of photon energies (20 eV~120 eV) to cover sufficient k_z–span as shown in Fig. S4. The periodicity along the k_z direction is hard to discern due to the complexity of the bulk bands (in addition to the surface states discussed in the Fig. 3a of the manuscript) around E_F (Supplementary Fig. 4a); but the constant energy contour at 200 meV below E_F shows clear periodicity of the feature at k_y=0 along the k_z direction (Supplementary Fig. 4b). The observed periodicity is expected from the bulk band nature of $MoTe_2$ and shows nice agreement with the calculated value from the lattice constant.

Supplementary Note 3: Spin polarization of the surface arc states

Similar to other topological surface states, the surface Fermi arcs should also possess non-degenerate spin texture. Thus to further demonstrate their non-trivial topological nature, we carried out spin-resolved photoemission experiments. The measurements were conducted at the FS and the dashed line in Supplementary Fig. 6a indicates the sampling direction that cuts through the Fermi

arcs (along k_x direction at $k_y=0.24$ Å^{-1}). The resulting polarized scattering intensity (Supplementary Fig. 6b and 6c) and the spin polarization asymmetry (Supplementary Fig. 6d and 6e) parallel to k_x and k_y directions are shown in Supplementary Fig. 6b~6e, clearly indicating the non-degenerate spin texture. Remarkably, while the spin polarization parallel to k_x direction (i.e. S_x, see Supplementary Fig. 6d) switches sign with positive and negative k_x values, the polarization parallel to k_y direction (i.e. S_y, see Supplementary Fig. 6e) have the same sign (though with less magnitude than S_x). Such observation of the non-degenerate spin structure is consistent with the non-trivial topological nature of the surface Fermi arcs, which will help understanding spin-related physical phenomena in $MoTe_2$. However, we notice that the spin polarization signal can also contributed from the SS which we cannot completely exclude, due to the relatively low angle and energy resolution (compared to regular ARPES) of the Spin-measurement (caused by the low spin-detection efficiency).

.
